# Corneal Pathology and Cataract: Combined Surgery or Sequential Surgery?

**DOI:** 10.4274/tjo.galenos.2020.04382

**Published:** 2021-02-25

**Authors:** Selma Özbek Uzman, Züleyha Yalnız Akkaya, Emrah Düzova, Evin Şingar, Ayşe Burcu

**Affiliations:** 1 University of Health Sciences Turkey, Ankara Training and Research Hospital, Clinic of Ophthalmology, Ankara, Turkey

**Keywords:** Combined surgery, sequential surgery, penetrating keratoplasty, open-sky cataract extraction, phacoemulsification and intraocular lens implantation

## Abstract

**Objectives:**

To compare our combined surgery (CS) and sequential surgery (SS) results.

**Materials and Methods:**

The files of 44 patients who underwent CS (penetrating keratoplasty [PK], cataract extraction, and intraocular lens [IOL] implantation) and 126 patients who underwent SS (cataract extraction and IOL implantation in a second session after PK) between January 2009 and December 2018 were evaluated retrospectively. One eye of the patients who were followed up for at least 1 year was included in the study. The two groups were compared in terms of indications, corrected distance visual acuity (CDVA), refractive results, complications, and graft survival.

**Results:**

In the CS and SS groups, the median age was 63 (30-79) and 43 (18-73) years (p<0.001) and the median follow-up time was 51 (13-152) and 64.5 (13-154) months (p=0.011), respectively. The most common PK indications were traumatic corneal scar (20.5%) and endothelial dystrophy (15.9%) in the CS group versus keratoconus (24.6%) and stroma dystrophy (17.5%) in the SS group. In the CS and SS groups, 50% vs 69% of patients had CDVA ≥0.4 (p=0.04); 45.5% vs 25.4% had CDVA (0.1-0.3) (p=0.04); and 54.5% vs 73% had spherical equivalent ≤±2.0 D (p=0.02). The most common postoperative complications were glaucoma (20.5% vs 15.9%, p=0.48) and allograft reaction (9.1% vs 23%, p=0.04). Graft survival rates were 95.2% vs 86.5% (p=0.10) at 1 year and 75.9% vs 68.9% (p=0.47) at 5 years, respectively.

**Conclusion:**

Over long-term follow-up, the groups were similar in terms of graft survival. For this reason, each patient must be evaluated separately whether to perform a combined or sequential surgery. Given the lower refractive error and higher expectation of final visual acuity, SS can be more advantageous especially in young patients.

## Introduction

Patients with cataract and corneal pathology that requires penetrating keratoplasty (PK) can be treated by combined surgery ([CS]: PK, cataract extraction, and intraocular lens [IOL] implantation in the same session) or sequential surgery ([SS]: PK followed by cataract extraction and IOL implantation in a second session).^1,2,3,4^

CS is particularly useful in cases where surgical trauma, postoperative inflammation, and drug therapy may cause progression of existing cataract, or in elderly patients with one eye or major health problems that necessitate faster visual recovery.^5,6,7,8^ In the combined approach, cataract surgery can be performed by phacoemulsification or open-sky extracapsular cataract extraction, depending on the severity of corneal opacity and the surgeon’s skill.^5,7,8,9^ The main disadvantages of CS are that in the open-sky technique, the eye is exposed to the outside environment for a longer time compared to PK alone, resulting in an increased risk of expulsive hemorrhage and endophthalmitis, and refractive errors are higher due to postoperative changes in keratometric values, anterior chamber depth, and axial length.^5,8,9,10,11,12,13,14,15^

SS is safer because it is performed in a closed system.^12,16,17^ Furthermore, more accurate IOL power calculation due to realistic keratometry, anterior chamber depth, and axial length measurements, placement of the incision on the steep axis during cataract surgery, and additional procedures such as compression sutures, arcuate keratotomy, or toric IOL implantation enable achievement of the most appropriate target refraction possible.^3,4,16,17^ The disadvantages of SS are endothelial loss and allograft reaction, the additional risk associated with a second anesthesia and surgery, delayed visual rehabilitation due to the time required for stabilization of keratometric values and suture removal, and higher cost.^3,4,16^

Advances in lamellar corneal surgeries have led to a reduction in PK surgeries and indications.^18,19,20,21,22,23^ However, PK still has a critical role in chronic bullous keratopathy, full-thickness corneal scars, and medically refractory microbial keratitis, in full-thickness perforations, and in large Descemet’s membrane ruptures during deep anterior lamellar keratoplasty.^3,4,18,19,20,21,22,23^ The visual and refractive outcomes of cataract surgery when combined or performed sequentially with PK and the effects of these procedures on graft survival are still controversial today.

Therefore, the aim of this study was to compare CS and SS in terms of indications, visual outcomes, complications, and graft survival.

## Materials and Methods

The study was approved by the local ethics committee and conducted in accordance with the principles of the 2013 revision of the Declaration of Helsinki. Informed consent forms were obtained from all patients before surgery. The medical records and surgical videos of 44 patients who underwent CS and 126 patients who underwent SS for various indications between January 2009 and December 2018 were retrospectively evaluated. Only the first operated eye of each patient was included in the study. Eyes followed up for less than 1 year were excluded. All donor corneas were obtained from the eye bank in our hospital. CS consisted of PK, open-sky extracapsular cataract extraction, and IOL implantation performed in the same session; in SS, phacoemulsification and IOL implantation were performed in a second session at least 6 months after PK. Axial length was measured using A-scan ultrasonography and corneal curvature was measured using Javal manual keratometer (Haag-Streit, Switzerland) or Scheimpflug topography (Pentacam; Oculus, Wetzlar, Germany). If the keratometry value could not be measured, a standard fixed keratometry value of 44 diopters was used.

In both groups, all surgeries were performed under general anesthesia by a single physician (A.B.). Before keratoplasty, hyperosmotic agents were administered intravenously to reduce vitreous pressure. In CS, a Flieringa ring was fixed to the sclera with 8/0 vicryl suture to prevent globe collapse. Taking into account the recipient’s horizontal and vertical corneal diameter, an area of the recipient cornea including the existing corneal pathology was excised using a vacuum trephine (Katena Products Inc., Denville, NJ, USA) and a donor cornea graft 0.50 to 0.75 mm larger than the recipient bed was cut from the endothelial side using a vacuum punch (Katena Products Inc., Denville, NJ, USA). The excision was then completed using right and left transplantation scissors. After “can opener” capsulotomy, hydrodissection and hydrodelineation were performed. The nucleus was removed using a vectis and the remaining cortex was manually aspirated using a Simcoe irrigation-aspiration cannula. After injection of ophthalmic viscoelastic, a polymethyl methacrylate posterior-chamber IOL was placed in the ciliary sulcus. The donor cornea was sutured to the recipient bed using interrupted sutures in eyes with a vascularized recipient bed and with continuous sutures in all others. At the end of the operation, keratoscopy was performed to adjust suture tension and corneal astigmatism.

In SS, phacoemulsification and IOL implantation were performed at least 6 months after PK. The anterior chamber was entered through a scleral tunnel incision made at the steep axis and continuous circular capsulorhexis followed by phacoemulsification were performed. The three-piece IOL was implanted in the capsular bag. The results of measurements obtained at least 3 months after surgery or suture removal were used in the analysis of refractive and visual outcomes.

The CS and SS groups were compared in terms of corrected distance visual acuity (CDVA), refractive outcomes, graft transparency, and complications.

### Statistical Analysis

SPSS for Windows version 16.0 (SPSS Inc. Chicago, USA) was used for statistical analyses. The Kolmogorov-Smirnov test was used to determine whether numerical data were normally distributed. Since all numerical data were non-normally distributed, descriptive statistics were represented as median (minimum-maximum). The Mann-Whitney U test was used to test the significance of the difference between the two medians. Qualitative variables were represented as frequency and percentage. P-values less than 0.05 were considered statistically significant.

## Results

In this study, the median age was higher in the CS group (p<0.001), while the follow-up period was longer in the SS group (p=0.011). The male to female ratio was similar in both groups (p=0.9) (Table 1).

The most common indications for PK were traumatic corneal scarring and endothelial dystrophy in the CS group and keratoconus and stroma dystrophy in the SS group (Table 2).

The pre- and postoperative visual and refractive outcomes of both groups are summarized in Table 3. The proportion of patients with CDVA ≥0.4 (Snellen) at final examination was significantly higher in the SS group (p=0.04), while the proportion of patients with a final CDVA of 0.1-0.3 (Snellen) was significantly higher in the CS group (p=0.04). CDVA was 1.6 (3-0.7) logMAR in the SS group before cataract surgery and increased to 0.4 (3.0-0) logMAR at 1 year after cataract surgery (p<0.001).

Median recipient diameter was 7.75 (7.25-8.00) mm in the CS group and 7.50 (6.00-8.50) mm in the SS group (p<0.001). Median donor diameter was 8.25 (7.75-8.50) mm in the CS group and 7.75 (6.50-9.00) mm in the SS group (p<0.001). Mean recipient and donor diameters were significantly larger in the CS group than the SS group (p<0.001). Visual and refractive outcomes of the groups according to median donor diameter are shown in Table 4.

Pre- and postoperative complications are shown in Table 5. Of the eyes that developed allograft reaction, 1 eye (2.3%) in the CS group and 9 eyes (7.1%) in the SS group did not respond to topical and systemic treatment (p=0.23). Of the eyes that developed postoperative glaucoma, 3 eyes (6.8%) in the CS group and 11 eyes (8.7%) in the SS group required surgical treatment (trabeculectomy/Ahmed Glaucoma Valve implantation) due to refractory IOP elevation (p=0.69).

One-year graft survival was 95.2% (42 of 44 eyes) in the CS group and 86.5% (109 of 126 eyes) in the SS group (p=0.10). Five-year graft survival was 75.9% (22 of 29 eyes) in the CS group and 68.9% (71 of 103 eyes) in the SS group (p=0.47).

## Discussion

When cataract is accompanied by corneal pathology, a choice must be made between combined or sequential surgery.^2,3,4^ There is ongoing controversy as to which method is more appropriate.^15,24,25^

In the literature, the most common indication for CS is reported to be Fuchs endothelial dystrophy (FED).^4,17^ In elderly patients with FED and patients with traumatic scarring, corneal pathology is often accompanied by cataracts that require surgery.^2,3,4,5,25^ In addition, existing cataracts may progress rapidly due to surgical trauma, inflammation, and corticosteroid therapy.^24,25^ Therefore, CS is recommended for patients whose cataracts are expected to impair vision to approximately 20/40 or lower.^24,25^

In our study, traumatic corneal scarring and endothelial dystrophy were the most common keratoplasty indications in the CS group, which had a higher median age than the SS group. Moderate to severe lens pathology was also detected in all of these patients. Combined cataract surgery with PK was preferred as it protects older patients from the increased risks of a second anesthesia due to comorbid systemic diseases and provides faster visual rehabilitation. The most common indications for keratoplasty in the SS group were keratoconus and stromal dystrophy. Lens pathology in these patients developed or progressed during follow-up.

In the literature, CDVA ≥20/40 has been reported in 38-64% of the cases after CS and in 64-100% of the cases after SS.^26,27,28^ In our study, preoperative CDVA was significantly lower in the CS group than the SS group due to the concomitant lens pathology requiring surgery in the combined group. When the groups were evaluated separately, CDVA increased significantly after both CS and SS compared to preoperative values. Although the two groups were found to be similar in terms of CDVA at the last examination, the proportion of patients with postoperative CDVA ≥0.4 was higher in the SS group (p=0.04). Consequently, a higher proportion of patients in the CS group had a CDVA of 0.1-0.3 (p=0.04). According to our results, although final visual acuity values seem numerically similar when compared with the significance test of difference between two medians, more patients achieved a visual acuity of at least 0.4 with SS. The lower proportion of patients who achieved a visual acuity of at least 0.4 after CS may be due to the fact that we did not exclude posterior pole and optic nerve diseases, which occur secondary to advanced age and trauma and may be more common in this group.

In addition to providing more accurate post-keratoplasty keratometry and topography measurements to use in IOL power calculation, SS also allows refractive errors associated with the keratoplasty to be corrected during cataract surgery.^3,4,5,8,11,29,30,31^ Although good anatomical outcomes are obtained with CS, refractive outcomes were not found to be equally favorable due to post-PK changes in keratometric values, axial length, and anterior chamber depth, which play a major role in IOL power calculation.^7,10,13,14,26,27^ Researchers have tried using various adjustments to deal with the changes in keratometric values after PK, but there is still no consensus on a default keratometric value.^10,11^ Therefore, keratometry measurements of the affected eye, those of the fellow eye if the affected eye cannot be measured, or a fixed keratometry value (44 D) can be used.^8,26,28,29,30,31,32^

In the literature, a target refraction of ±2.0 D was reported to be achieved in 26-68% of eyes after CS and 67-95% after SS.^3,7,10,14,16,26,28,32^ Consistent with the literature, the proportion of eyes that achieved a target refraction of ±2.0 D at the last postoperative examination was higher in the SS group (73%) than in the CS group (54.5%) due to the fact that keratometric and axial values could be measured more accurately (p=0.02). However, the proportion of eyes with refractive astigmatism <±3.0 D was similar in the CS group (52.3%) and SS group (62.7%) (p=0.22). The larger recipient bed to allow lens removal and the resulting use of larger diameter grafts in the CS group, and making a steep-axis cataract incision in the SS group may have had a role in reducing astigmatism in both groups.

Today, Descemet’s membrane endothelial keratoplasty has replaced PK in endothelial diseases with clear stromal tissue.^20^ Although CS consisting of cataract extraction and lens implantation together with endothelial keratoplasty provides more predictable refractive outcomes, PK is still indispensable in the presence of stromal scar.^17,19,33^ Some of the patients with endothelial dystrophy in our CS group underwent surgery before we transitioned to endothelial keratoplasty. Some also underwent penetrating surgery because endothelial keratoplasty was not a suitable option due to accompanying stromal opacity.

Sight-threatening intraocular complications such as expulsive hemorrhage, posterior capsule rupture, and vitreous loss are reported to be more common in patients undergoing CS compared to SS.^3,4,24,34,35,36,37^ These complications usually occur during the “open-sky” stage of the procedure, between the removal of the recipient cornea and implantation of the donor cornea. This dangerous period in which the globe remains open is prolonged in CS due to cataract extraction and IOL implantation, resulting in a higher frequency of these complications.^3,4,5,15,34,35,36,38^ Increased systemic blood pressure or Valsalva maneuver (cough) during this period might lead to posterior capsule rupture, vitreous prolapse, or pulmonary hemorrhage.^12,24,34^ In our study, the two groups were similar in terms of posterior capsule rupture and vitreous loss. Expulsive hemorrhage was not observed. This may be attributed to our general preoperative precautions such as systemic blood pressure control before and during surgery, proper positioning of the patient’s head, selection of an appropriate eyelid speculum, and using intravenous hyperosmotic agents to reduce vitreous pressure.

The incidence of glaucoma after PK is 13-38% in phakic cases and 42-89% in aphakic cases, with a mean of 33%. In addition, its incidence is 9-31% in the early postoperative period and 18-35% in the late postoperative period.^39,40,41,42,43^ Trabecular network collapse and angle distortion, decreased outflow resulting from extensive peripheral anterior synechiae in the late period due to postoperative edema and inflammation, and steroid sensitivity are important factors in glaucoma development.^39^ In patients at high risk for glaucoma, we take precautions such as selecting a graft diameter at least 0.5 mm larger than the recipient bed, making shorter suture passages, performing goniosynechialysis in patients with peripheral anterior synechiae, completely clearing residual viscoelastic from the anterior chamber, and discontinuing steroids as soon as possible postoperatively. This may explain why the rates of glaucoma and need for glaucoma surgery in both groups were similar and lower than those in the literature (CS: 20.5%, SS: 15.9%).

Infectious keratitis after keratoplasty is a rare but serious complication. In the literature, the incidence of graft infection after PK has been reported as 1.5-12.6% and the incidence of endophthalmitis as 0.1-0.7%.^3,7,8,44^ In our study, the incidence of graft infection was similar to the literature in both groups and no early endophthalmitis was observed.

Since cataract surgery causes 10% endothelial loss, graft failure can occur at rates of 13-21% with sequential cataract surgery performed after PK.^3,15,24,45,46,47,48^ In our study, the prevalence of endothelial failure in the SS group (9.5%) was lower than in the literature. This may be attributed to improved surgical techniques, advances in surgical microscopes and microsurgery, a better understanding of corneal metabolism, and reduced endothelial loss due to modern eye banking and donor tissue storage. Another reason may be the younger age of patients in the SS group, and with the general approach of preferring younger donors, the result is the transplantation of grafts with higher endothelial cell counts to recipient beds with keratoconus and stromal dystrophy that have normal endothelial cells. Although there is no significant endothelial cell loss after CS, the additional surgical interventions are reported to activate the immune response, which may be associated with an increase in graft rejection episodes due to increased inflammation.^15,24,28,47^ In our study, the incidence of allograft reaction was higher in eyes treated with SS (23%) than eyes treated with CS (9.1%) (p=0.04). However, rates of non-response to topical and systemic immunosuppressive therapy in the early period after allograft reaction were similar in both groups (CS: 2.3%, SS: 7.1%). The higher frequency of immune response in the SS group may be due to the inability of topical steroids to suppress postoperative inflammation, and the fact that the SS group had a lower median age and was a more immunologically active population.

Reported graft survival rates in the literature are 69-100% after CS and 79-91% after SS.^3,11,30,36,45,46,47,48,49^ In our study, the CS and SS groups showed similar 1-year (95.2% and 86.5%) and 5-year (75.9% and 68.9%, respectively) graft survival rates.

### Study Limitations

Limitations of our study were that the two groups differed in terms of keratoplasty indications, age, suturing technique, and graft diameter. In addition, other comorbid ocular diseases such as posterior segment disorders and glaucoma, which may have an effect on final CDVA, were not excluded. Strengths of our study are that the surgeries were performed by a single physician and we compared results from a long follow-up period of 5 years.

## Conclusion

CS is advantageous because it involves a single surgery and provides faster visual rehabilitation, as well as reduced anesthesia risks and surgery costs. This is important for older patients with serious health problems and provides a rapid increase in vision. However, SS seems to be more advantageous, especially for young patients, given the low refractive error and expectation of high final visual acuity. Factors such as visual expectations, need for rapid visual rehabilitation, systemic comorbidities, and anesthesia risk should be evaluated to select the most appropriate procedure for the patient.

## Figures and Tables

**Table 1:**
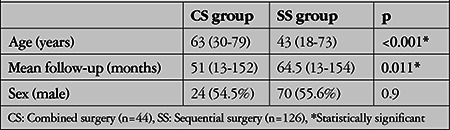
Demographic characteristics of the patients in the combined and sequential surgery groups

**Table 2:**
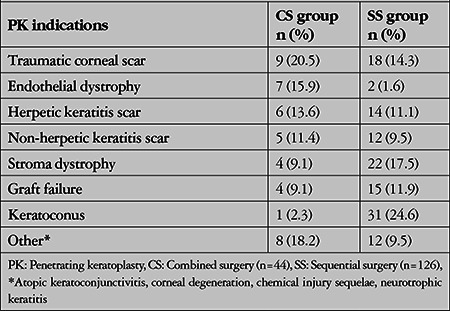
Indications for penetrating keratoplasty

**Table 3:**
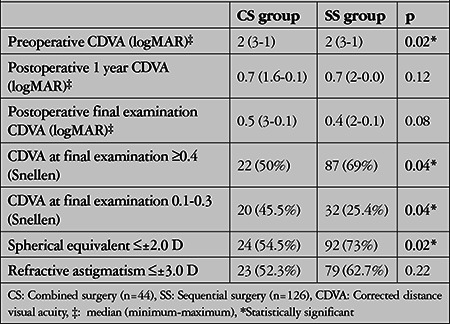
Visual and refractive results in the combined surgery and sequential surgery groups

**Table 4:**
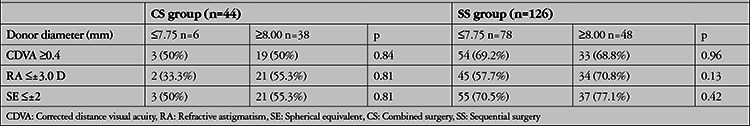
Visual and refractive results of the groups according to median donor diameter

**Table 5:**
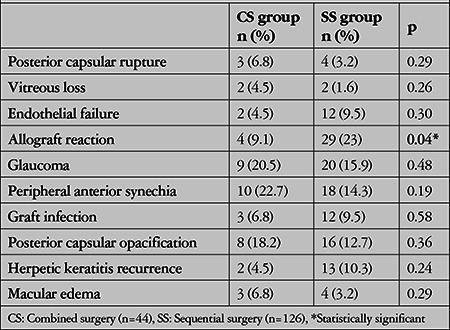
Postoperative complications
